# Radiomics‐based kidney lesion classification: Mitigating batch effect with nested combat harmonization

**DOI:** 10.1002/mp.18070

**Published:** 2025-09-01

**Authors:** Niloofar Ziasaeedi, Yannick Lemaréchal, Mohsen Agharazii, Venkata S. K. Manem, Philippe Després, Leyla Ebrahimpour

**Affiliations:** ^1^ Département de physique de génie physique et d'optique Université Laval Québec Québec Canada; ^2^ Centre de recherche du CHU de Québec Québec Québec Canada; ^3^ Centre de recherche de l'Institut universitaire de cardiologie et de pneumologie de Québec Québec Québec Canada; ^4^ Faculty of Medicine Université Laval Québec Québec Canada; ^5^ Department of Medical Physics Memorial Sloan Kettering Cancer Center New York New York USA

**Keywords:** kidney cancer, machine learning, radiomics

## Abstract

**Background:**

The increased use of CT imaging has elevated the incidental detection of renal masses, necessitating accurate differentiation between benign and malignant nodules. Radiomics offers potential for improved diagnostics; however, it is limited by variability in imaging parameters such as slice thickness, highlighting the need for effective harmonization techniques.

**Purpose:**

The purpose of this study is to conduct a comprehensive radiomics analysis, evaluating the impact of slice thickness in distinguishing between kidney cysts and tumors using machine learning techniques, thus contributing to more precise and effective patient management strategies.

**Methods:**

We utilized a publicly available dataset, KITS23, and extracted radiomic features from contrast‐enhanced computed tomography (CT) scans using the PyRadiomics library. The dataset consists of 599 cases, which were divided into training (60%) and testing (40%) cohorts to develop and validate predictive models. Six feature selection methods and ten machine learning classifiers were employed. Additionally, the Nested Combat harmonization technique was applied to address variations in imaging protocols across institutions.

**Results:**

We observed improvements in AUC values across various feature selection methods and classifiers after harmonization, with the highest AUC reaching 0.95. This represents significant enhancements in model performance, with mean AUC improvements ranging from 0.7% to 7.7% across different feature selection methods, bringing our results in line with, and in some cases surpassing, the AUCs reported in the literature.

**Conclusions:**

These findings underscore the potential of radiomics‐based machine learning models to enchance diagnostic accuracy and patient management in clinical practice. The use of harmonization techniques, such as, Nested Combat is crucial in achieving reliable and generalizable predictive models for renal oncology.

## 1 INTRODUCTION

The increased availability and utilization of advanced imaging modalities, notably computed tomography (CT), have led to a significant rise in the incidental detection of renal tumors.^1^ Among the incidental findings, cystic renal masses are frequently encountered during CT scans, with a prevalence ranging from 10% to 41% in adults,^2^ particularly in individuals over the age of 50.^3^ Athough the majority of these cystic masses are benign, a subset may harbor renal cell carcinoma (RCC).^4^ Accurate and prompt differentiation between benign and malignant renal tumors is essential for effective treatment. This necessitates comprehensive imaging evaluations and diagnostic methodologies to facilitate rapid and accurate treatment decisions.

In this context, radiomics analysis, which utilizes advanced computational techniques to extract quantitative features from medical images, holds promise for enhancing the characterization and differential diagnosis of renal masses. Radiomics also shows potential for improving cancer diagnosis and prognosis across various tumor types, including kidney,^5^ brain,^6^, ^7^ and lung^8^ cancers. Studies with CT phantoms have shown that variations in slice thickness and reconstruction algorithms significantly influence radiomic feature quantification.^9^, ^10^

A deeper comprehension of how imaging acquisition choices influence radiomic feature variability will bolster confidence in the validity of radiomic analyses, guide the standardization of imaging parameters, and augment the generalization and applicability of findings in this rapidly evolving field. Standardization methods that can be applied before or after extraction of radiomic features aim to mitigate these issues.^11^ ComBat,^12^, ^13^ as a post‐harmonization method, employs empirical Bayes estimation to correct imaging feature variations. Despite its effectiveness, ComBat has limitations, including assumptions about feature error distribution and the need for known batch effects.^14^, ^15^ A refined method, NestedComBat,^16^ iteratively applies ComBat to address multiple batch effects, providing a comprehensive solution for harmonizing diverse radiomic features. Recent advancements in radiomics analysis have shown promising results in diagnosing and characterizing renal masses. Yu et al.^17^ employed support vector machines (SVM) on 119 patients, achieving AUCs of 0.91 and 0.93, demonstrating the efficacy of texture features in distinguishing various RCC subtypes and oncocytoma. Nassiri et al.^18^ expanded on this, utilizing radiomics predictive models on 684 patients, reporting AUC values of 0.84 and 0.77 for distinguishing benign from cancerous lesions, emphasizing the importance of clinical variables. Coy et al.^19^ further reinforced the utility of radiomics, achieving an AUC of 0.85 in discriminating between malignant and benign lesions in 200 patients, highlighting the potential of computer‐aided design (CAD) systems. Erdim et al.^20^ contributed insights into ML‐based approaches, achieving an AUC of 0.915 and 91.7% accuracy in distinguishing renal cancer from benign masses, underscoring the importance of feature selection in enhancing performance. Collectively, these studies demonstrate the growing significance of radiomic analysis, leveraging advanced algorithms and imaging techniques to improve diagnostic accuracy in renal mass characterization. In this study, we conducted a comprehensive investigation utilizing machine‐learning‐based classifiers to leverage radiomic features extracted from contrast‐enhanced CT scans for distinguishing between benign and malignant kidney masses. Our analysis involved exploring various combinations of feature selection methods and classifiers. Subsequently, we applied the Nested Combat harmonization method specifically to address variations in slice thickness. To the best of our knowledge, this is the first study that systematically addresses the impact of slice thickness as a batch effect in radiomics‐based machine learning models for distinguishing kidney cysts and tumors. Furthermore, no prior studies have explored the effect of combining different feature selection methods with classifiers, nor have they examined how harmonization influences the performance of these combinations. Our findings highlight the importance of addressing slice thickness variability and demonstrate the significant improvements in model robustness and generalizability achieved through the use of harmonization techniques.

## 2 MATERIAL AND METHOD

### 2.1 Description of study population

In this investigation, we utilized the publicly accessible “Kidney and Kidney Tumor Segmentation Challenge 2023” (KiTS23) dataset in NIfTI format.^21^ This dataset comprises contrast‐enhanced CT scans and is the third iteration of the KiTS challenge, following previous editions held in 2019 and 2021. The KiTS23 cohort encompasses patients who underwent cryoablation, partial nephrectomy, or radical nephrectomy for suspected renal malignancy between 2010 and 2022 at the M Health Fairview medical center, Minneapolis, MN, United States. A retrospective analysis of these cases was performed to identify all patients who had received a contrast‐enhanced preoperative CT scan encompassing both kidneys entirely. The dataset consisted of 599 cases, where each case's most recent contrast‐enhanced preoperative scan, either in the corticomedullary or nephrogenic phase.

The KiTS23 dataset is available exclusively in NIfTI format, which does not preserve certain scanner‐specific parameters (e.g., manufacturer, reconstruction kernel). This highlights the importance of archiving original DICOM images whenever possible, as conversion to non‐DICOM formats such as MINC, NIfTI, Analyse, or BIDS may result in the loss of critical metadata embedded in DICOM objects particularly acquisition‐related parameters. Retaining complete metadata can facilitate more detailed investigations into imaging parameters and their effects on radiomic features.^22^

The dataset segmentation involved three primary classes. First, the “Kidney” category encompasses all parenchyma and non‐adipose tissue within the hilum. Second, the “Tumor” class refers to masses identified on the kidney that were identified as malignant after surgery. Lastly, the “Cyst” classification represents kidney masses determined to be cysts based on radiological findings or pathological examination, if available.

### 2.2 Feature extraction

PyRadiomics facilitates the processing and extraction of radiomic features from medical image data through a comprehensive collection of engineered hard‐coded feature algorithms. This open‐source python package allows for the extraction of radiomics features from various medical imaging modalities, including CT, PET, and MRI.^23^ In the initial step, CT scans, along with segmentation maps, are imported into the feature extractor for processing. In our analysis using PyRadiomics, we employ a set of predefined settings to standardize feature extraction from CT data. The preprocessing steps include resampling all scans to 1‐mm isotropic voxels using the B‐Spline interpolation method (sitkBSpline), ensuring uniform voxel size and improving the accuracy and comparability of radiomic features. To prevent edge artifacts during filtering with large sigma values, an additional padding of 10 mm is applied around the entire image, This padding ensures that image borders are not affected by the filtering process. Our analysis incorporates three image types: (1) Original, (2) Wavelet‐filtered, and (3) Laplacian of Gaussian (LoG)‐filtered images with sigma values of 1.0, 2.0, 3.0, 4.0, and 5.0. A low sigma highlights fine textures with rapid changes over short distances, while a high sigma emphasizes coarse textures with gradual gray‐level changes over larger distances. Feature classes extracted from the segmented masses inside the kidney, whether tumor or cyst include:

- Shape features describe the three‐dimensional geometrical properties of the segmented tumor, including volume, surface area, and sphericity.
- First‐order statistics summarize the distribution of voxel intensities within the region of interest, capturing measures such as mean, median, and skewness.
- Texture features, derived from matrices such as the Gray Level Co‐occurrence Matrix (GLCM), Gray Level Run Length Matrix (GLRLM), Gray Level Size Zone Matrix (GLSZM), and Gray Level Dependence Matrix (GLDM), quantify patterns and relationships between neighboring voxels, providing insights into the homogeneity, contrast, and complexity of the texture.

We also used a bin width of 25 for image discretization and apply a voxel array shift of 1000 to prevent negative values in the Hounsfield Unit (HU) scale. The bin width values were selected based on the first‐order range in the dataset to achieve a roughly equivalent number of gray levels compared to the bin count method.^24^ Out of the 1218 radiomic features extracted from the dataset, 100 were derived directly from the original CT scans. Among these, 14 were shape‐based, 18 were intensity‐based (first‐order), and 68 were texture‐based features. The remaining 1118 features were extracted from images processed with various filters, including Wavelet, LoG, Gradient, Square, Square‐root, and Exponential filters.

### 2.3 Feature harmonization

One of the primary limitations of radiomics is the absence of standardized image acquisition protocols. Contrast‐enhanced CT images, captured at various phases (plain, arterial, and venous) and with varying reconstructed slice thicknesses, can significantly impact the radiomics outcomes.^25^, ^26^ Given that slice thickness significantly impacts the quantification of radiomic features, with thinner (1.25 and 2.5 mm) and thicker (5 mm) slices yielding different results,^27^ we aimed to address variability introduced by different reconstruction process. To achieve this, we applied the ComBat harmonization technique,^12^, ^13^ specifically utilizing Nested ComBat.^16^ NestedComBat corrects for batch effects while preserving biological variability by adjusting for the hierarchical structure of the data. As described in its original publication,^16^ Nested Combat is a sequential harmonization method designed to accommodate multiple imaging parameters simultaneously, thus providing additional flexibility compared to standard Combat. Although prior work has shown that Nested Combat can achieve performance similar to standard Combat when reducing features with statistically significant differences (in part due to bimodal feature distributions), its ability to iteratively adjust for multiple batch effects makes it particularly promising for radiomic analyses that involve complex acquisition protocols. In the present study, the novelty lies in applying Nested Combat for the first time to differentiate kidney tumors from cysts from a dataset featuring heterogeneous slice thicknesses. This specific application addresses the recognized impact of slice thickness variability on radiomic feature stability, with potential for future expansion to incorporate other parameters such as reconstruction algorithms, scanner manufacturers, and contrast enhancement phases. The primary harmonization factor considered in this study is slice thickness, ensuring consistency across the dataset. By applying Nested Combat, we aimed to enhance the robustness and reproducibility of the radiomics features, enabling more reliable downstream analysis and classification. The dataset included twelve different slice thicknesses: 0.5 mm (*n* = 62), 1.0 mm (*n* = 86), 1.25 mm (*n* = 8), 1.5 mm (*n* = 5), 2.0 mm (*n* = 28), 2.5 mm (*n* = 59), 3.0 mm (*n* = 133), 3.75 mm (*n* = 5), 4.0 mm (*n* = 10), and 5.0 mm (*n* = 334). These variations in slice thickness introduced significant variability in the quantification of radiomic features, highlighting the necessity of harmonization techniques to ensure consistency across different imaging protocols. We employed a Python‐based approach to harmonize radiomic features using Nested Combat. Briefly, we categorized slice thickness values into bins to define the primary batch effect, combined them with relevant clinical covariates which included alcohol use status, gender, and age. The resulting harmonized dataset, which standardizes features across different acquisition conditions, served as input to our classification models for robust tumor‐versus‐cyst discrimination.

### 2.4 Feature selection

In radiomics, imaging data undergo transformation into high‐dimensional features. However, the high dimensionality of these features presents challenges, including the risk of overfitting and the need for effective feature selection methods. To address these challenges, we employed a two‐step feature selection process. In the first step, we used Lasso (Least Absolute Shrinkage and Selection Operator) regression with 10‐fold cross‐validation. Lasso is a regression technique that imposes a penalty on the absolute size of the coefficients, effectively shrinking some to zero and selecting only the most relevant features. This method reduces the dimensionality of the feature set and enhances the robustness and predictive power of our models. LASSO reduces the number of features and enhances the model's interpretability by eliminating less important variables. This dual benefit makes LASSO an effective tool for refining our models and ensuring that only the most relevant features are considered in distinguishing tumors from cysts in kidneys. To ensure adequate convergence of the LASSO algorithm across our dataset, we have set the maximum number of iterations to 10,000. Additionally, to achieve a precise exploration of the regularization path, we set Epsilon(In scikit‐learn's LassoCV, eps defines the ration of $\alpha_{min}$ to $\alpha_{max}$) to 0.001, allowing for a more detailed selection of the regularization parameter alpha.

Six feature selection methods were used in the analysis (Relief (Relief), Chi‐square (Chi), Mutual information maximization (MI), the classical Minimum redundancy maximum relevance (mRMR),^28^ Recursive feature elimination (RFE) and ANOVA F‐value(ANOVA). Relief is a feature selection algorithm that estimates feature importance based on the ability to distinguish between instances that are near each other. It works by repeatedly sampling an instance and evaluating the difference between the nearest instances of the same and different classes. The Chi‐square test evaluates the independence of two categorical variables. In feature selection, it measures the dependence between each feature and the target variable, selecting features that are most likely to be dependent on the target. MI is a method that selects features by measuring the amount of information shared between the feature and the target variable. Features with high mutual information are selected as they provide more information about the target. Classical mRMR aims to select features that are highly relevant to the target variable while being minimally redundant. It balances between maximizing the relevance of selected features and minimizing redundancy among them. RFE is an iterative feature selection method that fits a model and removes the least important feature(s) each time. This process continues until the desired number of features is reached, ensuring that the most significant features are retained. The ANOVA F‐value method selects features based on their F‐value from an Analysis of Variance (ANOVA) test. It evaluates each feature individually by measuring the ratio of variance between groups to the variance within groups, selecting features with the highest F‐values. By leveraging these feature selection methods, we aim to identify a subset of informative features that optimize the performance of our machine learning models while enhancing interpretability and generalization. Each method contributes unique insights into the underlying structure of the data, enabling us to make informed decisions regarding feature inclusion in our analysis. We selected these methods primarily due to their widespread use in the literature, as well as their simplicity and computational efficiency.

### 2.5 Classification

Classification in the field of machine learning often involves supervised learning, where the task is to learn a function from training data with annotations.^29^ Within the training dataset, a multitude of instances is present. Each instance comprises an input vector containing features alongside a corresponding desired output value, categorized as either “Tumor” or “Cyst”. The classification algorithm analyzes this training data to formulate a hypothesis. This hypothesis, once established, serves as the foundation for predicting the labels of observations that have not been previously encountered. Ten different machine learning methods were used to build the prediction model: Logistic Regression (LR), Random Forest (RF), SVM, Gradient Boosting (GB), Decision trees (DT), Bagging (BAG), Bayesian (BY), K Nearest neighbors (KNN), Neural networks (NN), and Adaboost (AD). These models were chosen for their diverse way of handling classification tasks, enabling a comprehensive evaluation of the feature sets' effectiveness in distinguishing tumors from cysts in kidneys. The classification models were implemented using the scikit‐learn package in Python, which provides a robust and user‐friendly framework for applying a wide range of machine learning algorithms. This streamlined the integration of various predictive modeling techniques into our analysis pipeline, enhancing the efficiency of our diagnostic assessments.

Each model was tuned with a grid search: LR (C = $10^{-3}$–$10^{3}$), RF (50–150 trees), SVM (C=0.1–10, linear/*rbf*, γ=scale/auto), GB (50–200 trees, learning‐rate: 0.001–0.1), AdaBoost (50–150 estimators, learning‐rate: 0.01–1.0), BAG (10–100 estimators), MLP (three hidden‐layer topologies), KNN (1–8 neighbours, three distance metrics: Euclidean, Manhattan, or Cosine), DT (maximum depth: 4–20, minimum samples per split: 2–10)

For each classifier, the combination of hyperparameters that maximized the mean area under the ROC curve (AUC) across all cross‐validation folds was selected. The model was then retrained on the entire training set using this optimal configuration before evaluation on the validation cohort. This procedure ensures that both variance and bias are appropriately managed while enhancing each classifier's predictive potential.

The classifiers underwent training via repeated (2 repetitions) 10‐fold cross‐validation on the training cohort. Subsequently, their predictive performance was assessed in the validation cohort using the area under the ROC curve (AUC).

### 2.6 Analysis workflow

**FIGURE 1:**
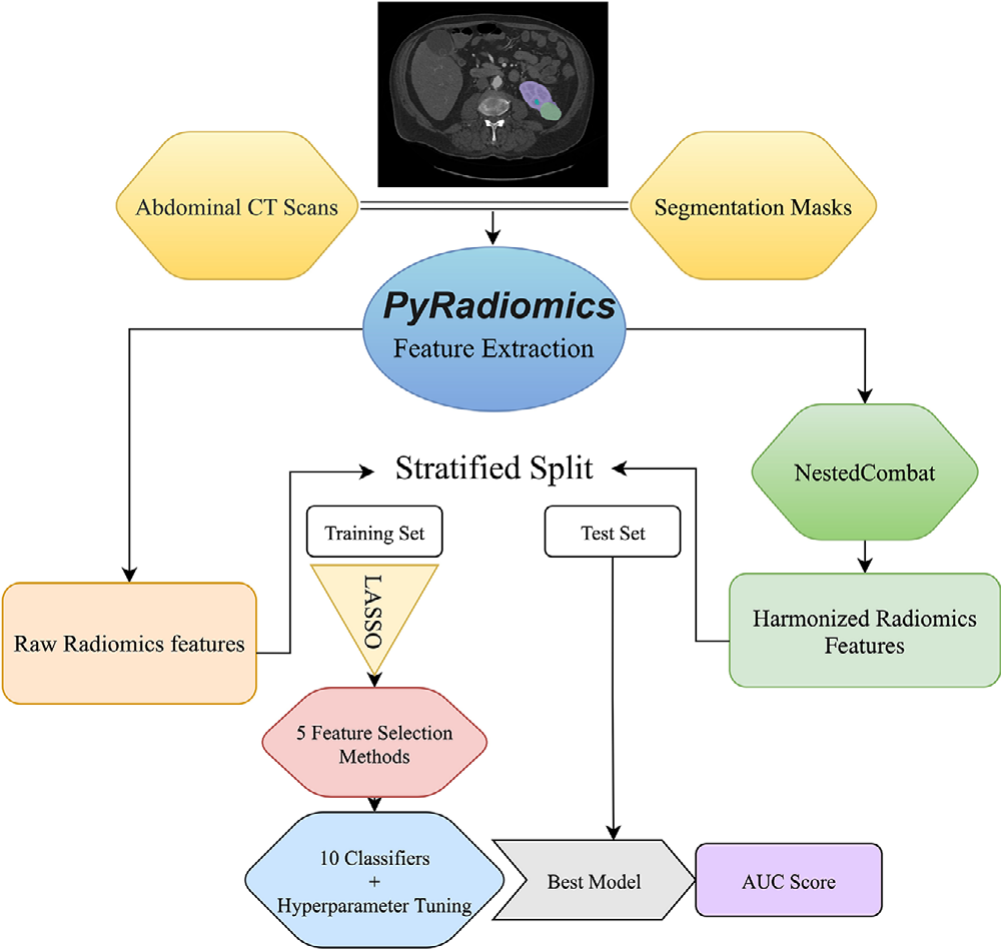
Analysis Workflow. Schematic of our entire pipeline from image acquisition through Nested ComBat harmonization and classifier evaluation.

An overview of the analysis workflow is shown in Figure 1. This study used 599 patients from the KITS23 dataset, establishing a comprehensive cohort for analysis. A 60%–40% split was applied to the dataset for training and testing, using StratifiedShuffleSplit to ensure the same proportion of tumors and cysts in both the training and testing sets, respectively. Utilizing the PyRadiomics library, radiomic features were extracted from CT images containing annotated lesions, enabling detailed characterization of tumor and cyst classes. All features in the training and testing cohorts were normalized to have a mean of zero and a standard deviation of one.

We conducted analyses using the original features extracted from PyRadiomics, as well as after applying Nested ComBat. The LASSO algorithm, which was optimized using a grid search using a 10‐fold cross‐validation, was employed to streamline feature selection and reduce dimensionality within the training dataset. Model development involved the integrating of six feature selection methods and ten ML algorithms. The analysis of different feature selection and classification methods was conducted by varying the number of selected features. We incrementally selected three to fifty features for each combination. Each of the classifiers was then evaluated against these subsets of selected features using area under the ROC curves (AUC). Hyperparameter tuning was conducted via a 10‐fold cross‐validation technique with GridSearchCV, optimizing model parameters and mitigating overfitting. Finally, the best‐performing models were evaluated on the test dataset using the area under the curve (AUC) metric, providing a robust assessment of classifier performance 1.

## 3 RESULTS

In our study, we evaluated all the trained models to identify the optimal combination of feature selection methods and classification techniques using a dataset of 295 lesions. The training cohort included 66.5% males and 33% females, with an average age of 60 years and a mean BMI of 31. The test cohort had similar demographics, with 61.7% males, 38.3% females, an average age of 61 years, and a mean BMI of 31. Additional characteristics such as smoking status, alcohol use, and diabetes prevalence were also comparable between the two cohorts. The demographic details of the train and test cohorts in this study is presented in Table 1. We assessed the comparability of the training and test cohorts using statistical tests. For continuous variables, *t*‐test showed no significant differences in age (*p* = 0.656) or BMI (*p* = 0.871). Chi‐Square tests for categorical variables also indicated no significant differences in gender (*p* = 0.791), smoking history (*p* = 0.707), alcohol use (*p* = 0.870), or diabetes status (*p* = 1.000). These results confirm that the training and test cohorts are statistically similar in key demographic and clinical characteristics.

**TABLE 1:** Cohort Characteristics.

Characteristic	Category	Training cohort	Test cohort
**Gender**	Male	66.5% (294)	61.7% (182)
	Female	33% (146)	38.3% (113)
**Age**	Mean ± SD	60.5 ± 13.7	60.9 ± 13.7
**BMI**	Mean ± SD	30.9 ± 7	30.9 ± 7
**Smoking**	Never smoked	46.4% (205)	44.7% (132)
	Previous smoker	38.2% (169)	40.7% (120)
	Current smoker	14.5% (64)	13.5% (40)
**Alcohol**	Never or not in the past 3 months	39.4% (174)	44.7% (132)
	Current users (more than two daily)	4.3% (19)	7.0% (21)
**Diabetes**	Yes	19.7% (87)	20.7% (61)

### 3.1 Performance analysis prior to harmonization

**FIGURE 2:**
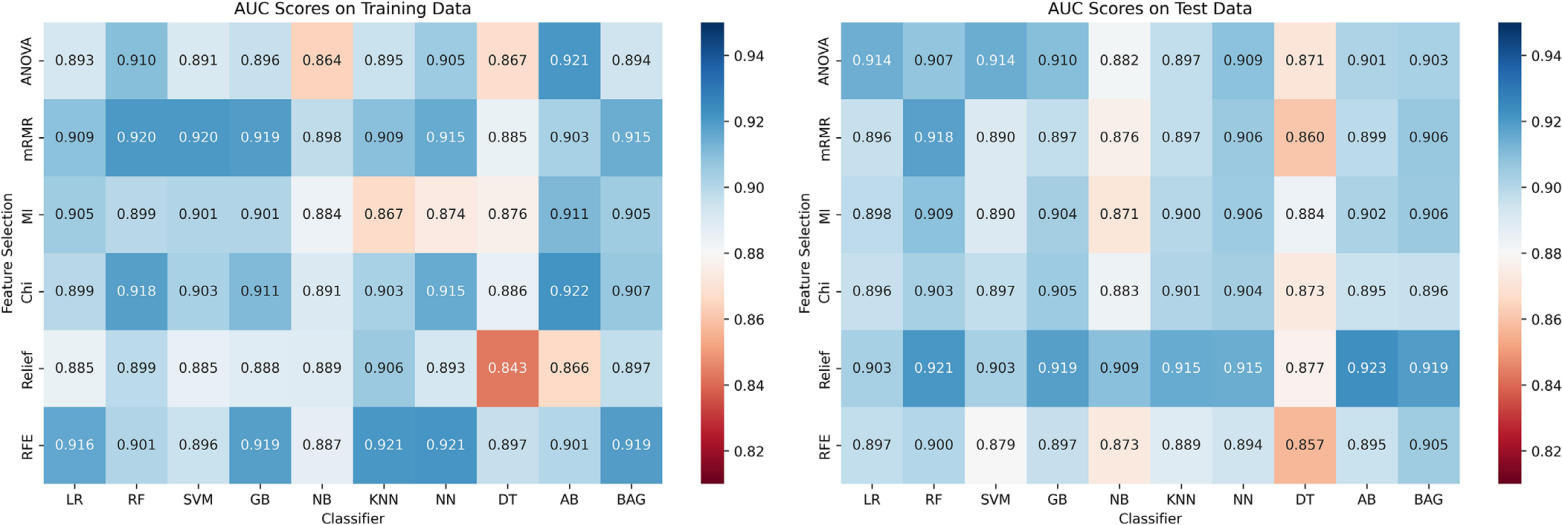
AUC scores for different feature selection methods and different classification methods on the training (left) and test data(right). AUC, area under the curve.

Initially, the models were evaluated based on their AUC scores without harmonization. The results, as presented in Figure 2 indicate varied performance across different combinations of feature selection methods and classifiers. The AUC scores varied from a minimum of 0.85 to a maximum of 0.92. Among the various combinations tested, the following three achieved the highest AUC scores: Relief and AdaBoost (AB) achieved the highest AUC of 0.92±0.06, Relief and RF also performed well with an AUC of 0.92±0.04 and ANOVA and LR delivered robust performance with an AUC of 0.91±0.03. The median AUC score across all models was 0.90.

### 3.2 Impact of harmonization on model performance

**FIGURE 3:**
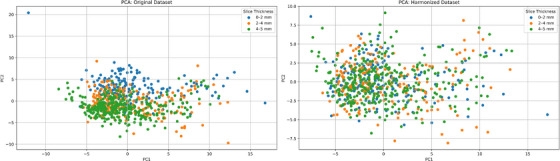
Principal component analysis (PCA) of PyRadiomics features from CT scans with different slice thicknesses before (left) and after (right) Nested ComBat harmonization.

We assessed the predictive function of radiomics with and without harmonization. We employed principal component analysis (PCA) to aid in visualizing the radiomic feature dataset. PCA was conducted to demonstrate the impact of slice thickness before and after applying Nested Combat. Additionally, PCA was performed separately for tumor and cysts after Nested Combat harmonization to illustrate the distinct clustering of these classes. Before harmonization of the data, the scatter plot on the left (Figure 3) shows distinct clusters based on slice thickness categories (0‐2, 2‐4, and 4‐5 mm). This clustering signifies that the radiomic features are significantly influenced by the thickness of the CT scan slices, potentially introducing batch effects that could impact the predictive accuracy and generalizability of models trained on this data.

After the harmonization process, the scatter plot on the right (Figure 3) shows a marked reduction in the distinctiveness of clusters based on slice thickness. The features appear more uniformly distributed, indicating that NestedComBat has effectively mitigated the slice thickness‐related variations. This results in a more integrated dataset with significantly reduced technical variability, enhancing the accuracy and consistency of predictive modeling by ensuring that outcomes are reflective of biological variations rather than artifacts of data acquisition.

**FIGURE 4:**
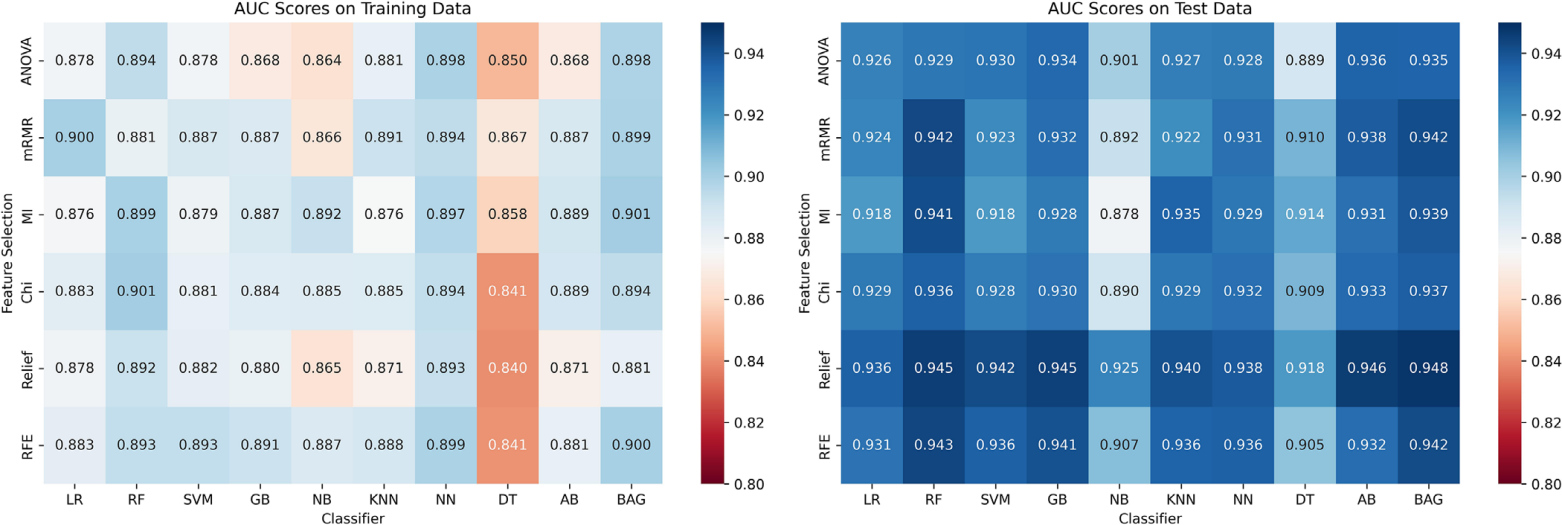
AUC scores for different feature selection methods and different classification methods on the training (left) and test data (right) after Nested ComBat harmonization. AUC, are under the curve.

**FIGURE 5:**
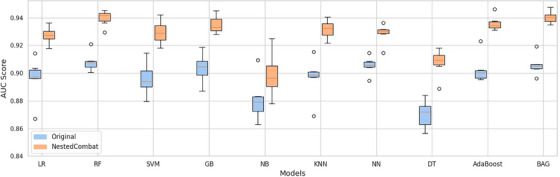
Boxplots of AUC distributions across feature selection methods for each classifier, before and after Nested ComBat harmonization. AUC, are under the curve.

After harmonization, we observed a noticeable improvement in the AUC scores across all combinations of feature selection methods and classifiers (Figure 4). Relief and BAG achieved the highest AUC of 0.94±0.04, relief and RF, GB, and AB also showed excellent performance with an AUC of 0.94±0.03. The median AUC score improved to approximately 0.93, reflecting a more robust central performance across all model combinations. The increase in both the median and maximum AUC scores post‐harmonization signifies that the models are not only performing better on average but also reaching higher peaks of performance. Figure 5 illustrates the distribution of AUC scores for various classifiers across different feature selection methods before and after applying NestedComBat harmonization. Each classifier shows a general improvement in AUC scores post‐harmonization and the median AUC scores increase for all classifiers, indicating a central tendency towards higher performance after harmonization. There is a noticeable reduction in the spread of AUC scores post‐harmonization across most classifiers. This reduction in variability (narrower interquartile ranges) suggests that models become more consistent in their predictive accuracy after the batch effects are mitigated. The presence of fewer outliers in the post‐harmonization results further indicates improved model stability and reliability. Classifiers such as BAG and AdaBoost show significant improvement and reach higher AUC scores, suggesting that these models particularly benefit from the harmonization process. This might be due to their ensemble nature, which could be more sensitive to the variances introduced by batch effects. The median increase ranges from approximately 1.79% for Naive Bayes to as high as 4.39% for DT. The range of percentage increases for each classifier further illustrates the variable impact of NestedComBat across different modeling techniques and feature selection strategies. For example, LR exhibits increases ranging from 1.3% to a notable 7.4%, indicating significant variability in how different feature selection methods respond to harmonization. Similarly, KNN shows a wide range of increases from 2.7% to 7.7%, underscoring the potential for substantial performance gains depending on the feature selection method used.

In addition to the qualitative improvement illustrated by the PCA (Figure 3), we conducted a quantitative assessment of the trained models before and after harmonization. For each classifier, we computed six AUC scores using six different feature selection methods, and performed a paired *t*‐test comparing these values before and after harmonization. This yielded one t‐statistic and one *p*‐value per classifier, allowing us to evaluate the effect of harmonization while accounting for dependency across feature selection methods. As summarized in Table 2, NestedComBat harmonization led to significant gains in predictive performance across all evaluated classifiers. Specifically, the mean AUC values increased by an average of 3% (range: 1.6%–3.7%), accompanied by robust t‐statistics (ranging from 3.98 to 19.27) and *p*‐values consistently below 0.05. The most pronounced improvement was observed for DT, which showed a mean difference of 0.0371 in AUC. To further validate these findings, we also conducted a non‐parametric Wilcoxon signed‐rank test, which does not assume normality and is particularly suitable for small sample sizes. Notably, the efficiency of the Wilcoxon signed‐rank test relative to the paired *t*‐test has been shown to be approximately 95% in small‐sample scenarios,^30^ supporting its reliability in our setting. The Wilcoxon test yielded identical *p*‐values (p=0.0312) across all classifiers, confirming the statistical significance of the observed improvements in AUC after harmonization. This consistency reflects the uniform positive shift in model performance and provides additional evidence for the robustness of our results. These enhancements underscore the effectiveness of NestedComBat in mitigating batch effects related to slice thickness variability, thereby reinforcing model stability and accuracy.

**TABLE 2:** Summary of statistical analysis for AUC scores before and after harmonization.

Classifier	AUC Before	AUC After	Mean difference	*t*‐Statistic	*p*‐Value
LR	0.90	0.93	0.026	7.12	$8.5\times 10^{-4}$
RF	0.91	0.94	0.030	9.31	$2.4\times 10^{-4}$
SVM	0.89	0.93	0.033	6.04	$1.8\times 10^{-3}$
GB	0.90	0.93	0.030	9.16	$2.6\times 10^{-4}$
NB	0.88	0.90	0.017	3.98	$1.05\times 10^{-2}$
KNN	0.90	0.93	0.032	9.23	$2.5\times 10^{-4}$
NN	0.90	0.93	0.027	8.46	$3.8\times 10^{-4}$
DT	0.87	0.91	0.038	7.56	$6.4\times 10^{-4}$
AB	0.90	0.93	0.033	13.42	$4.0\times 10^{-5}$
BAG	0.90	0.94	0.035	19.27	$1.0\times 10^{-5}$

*Note*: “AUC Before” and “AUC After” denote the mean AUCs across all feature selection methods before and after harmonization, respectively.

Abbreviation: AUC, area under curve; BAG, Bagging; DT, decision tree; GB, gradient boosting; KNN, K Nearest neighbors; LR, logistic regression; NB, NN, neural network; RF, Random Forest; SVM support vector machines.

We also evaluated how NestedComBat impacted the models' ability to fit the training data. Despite slight to moderate declines in training‐set AUC for some classifiers (e.g., a decrease of 1.3% for Naive Bayes up to 3.4% for BAG), the models still maintained strong performance on the training set overall. This reduction likely indicates that batch‐specific artifacts—particularly those tied to slice thickness—were being “unlearned” by the model. Indeed, the corresponding improvements in test‐set AUC confirm that harmonization successfully enhanced model generalizability (see Table 2). Hence, the slight decrease in training‐set fit, coupled with a notable gain on unseen data, underscores the effectiveness of Combat in mitigating overfitting to reconstruction‐dependent features.

## 4 DISCUSSION

This study investigated the effectiveness of Nested Combat harmonization in addressing batch effects due to slice thickness variations within a radiomic dataset derived from CT scans of kidneys. By employing a variety of classifiers and feature selection methods, we assessed the impact of this harmonization on the predictive accuracy as evidenced by changes in the AUC scores before and after applying the harmonization technique. Based on the performance analysis prior to harmonization, the models in this study achieved a maximum AUC of 0.923, particularly with the combination of Relief and AdaBoost, which is comparable to or exceeds the AUCs reported in earlier studies, such as Coy et al. and Yu et al., where AUCs ranged from 0.850 to 0.959 (Table 3).^17^, ^19^ The application of the Nested Combat harmonization technique further enhanced the robustness and reliability of the models. By addressing variability introduced by slice thickness, Nested Combat significantly improved AUC values across various feature selection and classifier combinations. The maximum AUC improved from 0.92 before harmonization to 0.94 after applying the harmonization process. This improvement strongly suggests that slice thickness variability was at least one major contributor to model performance drops. This highlights the importance of harmonizing radiomic features to ensure consistent and reproducible results, especially in multicenter studies where imaging protocols can vary widely.

The observed improvements in AUC post‐Nested Combat application indicate that the AUC achieved on the test data is more reliable and robust, reflecting the effectiveness of Nested Combat in mitigating unwanted variability and harmonizing the datasets. Additionally, it's worth noting that the dataset only included slice thickness information. The inclusion of additional variables such as kernel and manufacturer could potentially yield more comprehensive and informative results, enhancing the overall performance and interpretability of the predictive models. The comparison of mean AUC changes before and after Nested Combat application reveals that certain classifiers, such as LR and SVM, benefited more from harmonization than others. This underscores the need for a tailored approach when selecting classifiers and harmonization techniques based on the specific characteristics of the dataset.

Future work could explore the impact of additional imaging parameters, such as kernel and manufacturer information, on model performance. In parallel, we plan to investigate the interpretability of the most informative radiomic features identified across different models and feature selection techniques, both before and after harmonization, to evaluate whether they correspond to clinically or biologically meaningful patterns. Incorporating these variables could provide a more comprehensive understanding of how different imaging factors influence radiomic features and model outcomes. Additionally, integrating clinical variables alongside radiomic features could further enhance the predictive power and clinical applicability of the models. Furthermore, experimenting with different radiomics feature extraction tools may offer valuable insights, as various tools may capture different feature sets or handle preprocessing differently, potentially improving the robustness and generalizability of the models.

**TABLE 3:** Summary of previous studies: Machine learning methods and AUCs.

Author	Machine learning method	AUC
Coy et al.	In‐house developed software	0.850–0.959
Yu et al.	SVM	0.91–0.93
Erdim et al.	RF	0.905
Uhlig et al.	RF	0.83
Yap et al.	RF and AD	0.68–0.75
Nassiri et al.	RF and AD	0.84

Abbreviation: AUC, area under curve.

## 5 CONCLUSION

Our study highlights the critical impact of slice thickness on radiomics‐based machine learning models in distinguishing between kidney cysts and tumors. By applying the Nested Combat harmonization technique, we effectively addressed the batch effects introduced by varying slice thickness, which significantly improved the consistency and generalizability of our predictive models. Although our models achieved AUC values comparable to those reported in the literature, the primary advancement of our study lies in the improvement of results through harmonization, making the models more robust across different imaging protocols. This emphasizes the potential of radiomics analysis, when harmonized for acquisition‐related variability, as a reliable and non‐invasive diagnostic tool in renal oncology. Moving forward, incorporating additional clinical variables and imaging parameters, such as reconstruction kernel and manufacturer information, could further enhance the performance and interpretability of predictive models, contributing to the development of more comprehensive diagnostic tools for renal mass characterization.

## CONFLICT OF INTEREST STATEMENT

The authors declare no conflict of interest.
